# Comparison of real-life data of abiraterone acetate and enzalutamide in metastatic castration-resistant prostate cancer

**DOI:** 10.1038/s41598-021-93659-x

**Published:** 2021-07-08

**Authors:** Ayşe Demirci, Cemil Bilir, Burcu Gülbağcı, İlhan Hacıbekiroğlu, İbrahim V. Bayoğlu, İrem Bilgetekin, Sinan Koca, Havva Y. Çınkır, Nadiye Akdeniz, Deniz Gül, Ceyhun Varım, Umut Demirci, Berna Öksüzoğlu

**Affiliations:** 1 Department of Medical Oncology, Sakarya University Training and Research Hospital, Adnan Menderes caddesi, Sağlık Sokak, No: 195-54000, Adapazarı/Sakarya, Turkey; 2 Department of Medical Oncology, Dr Abdurrahman Yurtaslan Ankara Oncology Training and Research Hospital, Ankara, Turkey; 3 Department of Medical Oncology, Istanbul Medeniyet University Göztepe Training and Research Hospital, Istanbul, Turkey; 4 Department of Medical Oncology, Gaziantep University Faculty of Medicine, Gaziantep, Turkey; 5 Department of Medical Oncology, Adıyaman Training and Research Hospital, Adiyaman, Turkey; 6 Department of Urology, Sakarya University Training and Research Hospital, Sakarya, Turkey; 7 Department of Internal Medicine, Sakarya University Training and Research Hospital, Sakarya, Turkey; 8 Department of Medical Oncology, Memorial Ankara Hospital, University of Usküdar, Ankara, Turkey

**Keywords:** Prostate cancer, Outcomes research, Hormonal therapies

## Abstract

To compare enzalutamide (E) and abiraterone acetate (AA) in terms of efficacy, survival and to characterize prognostic factors affecting survival in metastatic castration-resistant prostate cancer (mCRPC) patients. A total of 250 patients treated with E or AA in 5 centers were included. The number of patients with no prostate specific antigen (PSA) decline was higher in the AA group than that in the E group, and the proportion of patients with a PSA decline of ≥ 50% was higher in the E group (p = 0.020). Radiological progression free survival (rPFS) and overall survival (OS) were significantly longer in the E group when compared to that in the AA group (p < 0.001 and p = 0.027, respectively). In the E group, rPFS was significantly longer than that in the AA group in both pre- and post-docetaxel settings (p = 0.010 and p = 0.003, respectively). OS was similar in the pre-docetaxel setting; but in the post-docetaxel setting, E group had a significantly longer OS than the AA group (p = 0.021). In the multivariate analysis performed in the whole patient group, we found that good prognostic factors for rPFS were E treatment, being ≥ 75 years and a PSA decline of ≥ 50% while there was no factor affecting OS. With longer OS and PFS, E seems to be more suitable for mCRPC patients in the post-docetaxel setting than AA.

## Introduction

Prostate cancer is the second most common cancer in men worldwide^1^. With the increasing use of screening tests, the majority of patients are at the local or locoregional stage at the time of diagnosis^2^. Androgen deprivation therapy (ADT) alone or in combination with other options is the main treatment for metastatic prostate cancer^3^. The majority of patients with advanced disease eventually progress while on ADT; then the condition is called castration-resistant prostate cancer (CRPC). Other treatment options besides ADT for metastatic CRPC (mCRPC) patients are chemotherapy (CT) (docetaxel, cabazitaxel), androgen synthesis inhibitors, androgen receptor blockers and Radium 223 radionuclide therapy^3^.

Abiraterone acetate (AA) and enzalutamide (E) are two main androgen receptor axis targeted agents used for the treatment of mCRPC^4,5^. Several pilot studies have shown that both drugs contribute significantly to overall survival (OS). After COU AA 301 and AFFIRM studies, AA and E were endorsed in the post-docetaxel setting. With the positive results obtained in COU AA 302 and PREVAIL, AA and E were approved in the pre-docetaxel setting.

The questions in front of us are the selection of patients we should use AA or E, and which one is advantageous in terms of efficacy and safety. Moreover, which one should be preferred before and after CT? Although the positive results of the phase III studies were achieved, we need real-world data with the results of larger patient groups to answer these questions more clearly. There is no head-to-head comparative phase III study related to E and AA. In a study on the simultaneous use of E and AA, it was concluded that the combination therapy had a manageable safety profile without significant drug-drug interaction; nevertheless it is not known whether the combination therapy is superior to the single agent therapy^6^. AQUARIUS, an observational, prospective study, which evaluated patient-reported outcomes in mCRPC patients who were treated with AA or E, suggested that AA was more advantageous than E in terms of fatigue and cognitive functions^7^.

Herein we aimed to compare E and AA in terms of baseline patient characteristics, efficacy and survival in mCRPC patients. Additionally, we analyzed prognostic factors affecting radiological progression free survival (rPFS) and OS in all patients.

## Materials and method

### Data collection

This study was designed retrospectively. A total of 250 patients diagnosed with mCRPC who were treated with E or AA between 2012 and 2020 in 5 centers were included in our study. All participating centers were from Turkey and included the following: Sakarya University Training and Research Hospital, Ankara Oncology Training and Research Hospital, Medeniyet University Göztepe Training and Research Hospital, Gaziantep University Faculty of Medicine, and Adıyaman University Training and Research Hospital. The patients were treated with E at a dose of 160 mg daily or AA at a dose of 1000 mg daily with prednisolone 10 mg daily until disease progression, death, or unacceptable toxicity. All patients, except those who had bilateral orchiectomy, continued to use ADT with serum testosterone levels 50 ng/dL (≤ 2.0 nmol/L).

Due to the retrospective design of our study, initial pain status in patients was unknown. Also, data on other prognostic factors such as albumin, LDH and hemoglobin could not be provided due to missing data. Since E or AA are not reimbursed in our country, no patients with hormone sensitive prostate cancer (mHSPC) received these treatments. For similar reasons, no patients received sequential AA and E or E and AA treatments during the data collection period.

### Clinical assessment

Prostate Cancer Working Group 2 (PCWG-2) criteria, death, or unacceptable toxicity were used to define disease progression. Prostate specific antigen (PSA) response was evaluated according to PCWG-2 criteria at the 12th week. The definition of mCRPC was biochemical or radiological progression, in accordance with the criteria of the PCWG, in patients with blood testosterone levels < 50 ng/dL. Patients who did not have mCRPC or those who received both E and AA were excluded from the study.

rPFS was defined as the time from the date of initiation of E or AA until the date of radiological progression. Patients were regularly followed up at 3-month intervals using thorax and abdomen computed tomography or abdominal ultrasonography and chest X-ray, and/or bone scintigraphy and/or Gallium-68 prostate specific membrane antigen positron emission tomography examinations. OS was defined as the time from the date of initiation of E or AA to the date of death from any cause. Increased or stable PSA levels at 12 weeks after E or AA initiation was defined as *No decline*, and a decline in the PSA level was grouped as < 50%* PSA decline* and ≥ 50%* PSA decline,* according to the decline rate. Radiological response rate (rRR) was evaluated in accordance with the Response Evaluation Criteria in Solid Tumors 1.1.

### Statistical analysis

Statistical analyses were performed using the IBM Statistical Package for the Social Science Statistics for Windows, version 22.0 (IBM Corp., Armonk, MY, USA). The variables were investigated using the Kolmogorov–Smirnov test to determine whether or not they were normally distributed. The continuous variables were expressed as mean ± standard deviation (for normally distributed variables) or median and interquartile range (IQR) (for not normally distributed variables). The Chi-square test or the Fisher’s exact test was used to compare the proportions in two groups. The Mann–Whitney U test was used to compare median PSA and median follow up time and the Student’s t-test was used to compare mean age. The Kaplan–Meier method was used to estimate survival. The log-rank test was used to identify the univariate effects of treatments and other factors on rPFS and OS of mCRPC patients. Possible factors associated with survival outcomes (p ≤ 0.250) in univariate analysis were selected for testing in multivariate models. The independent predictors of survival were determined with multivariate Cox regression models. A 5% type-I error level was used to infer statistical significance.

### Ethics approval

The study protocol was approved by the ethics committee of Sakarya University Medical Faculty and was conducted in accordance with the principles of the Declaration of Helsinki (05.03.2020-71522473/050.01.04/43). Given the retrospective study design, the need for informed consent was waived.

## Results

**Figure 1:**
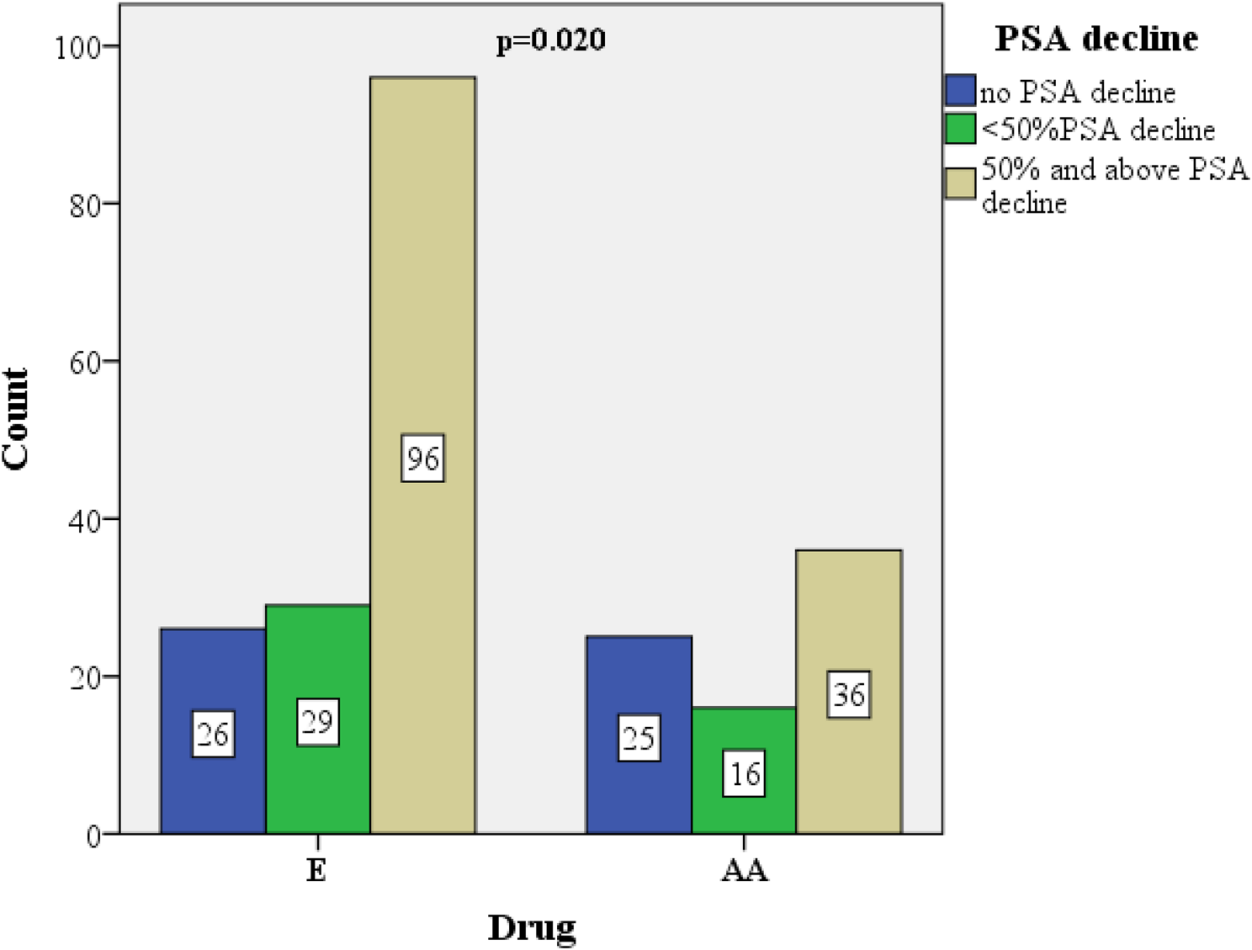
Comparison of PSA decline levels between drugs. *PSA* prostate-specific antigen *E* enzalutamide, *AA* abiraterone acetate.

**Figure 2:**
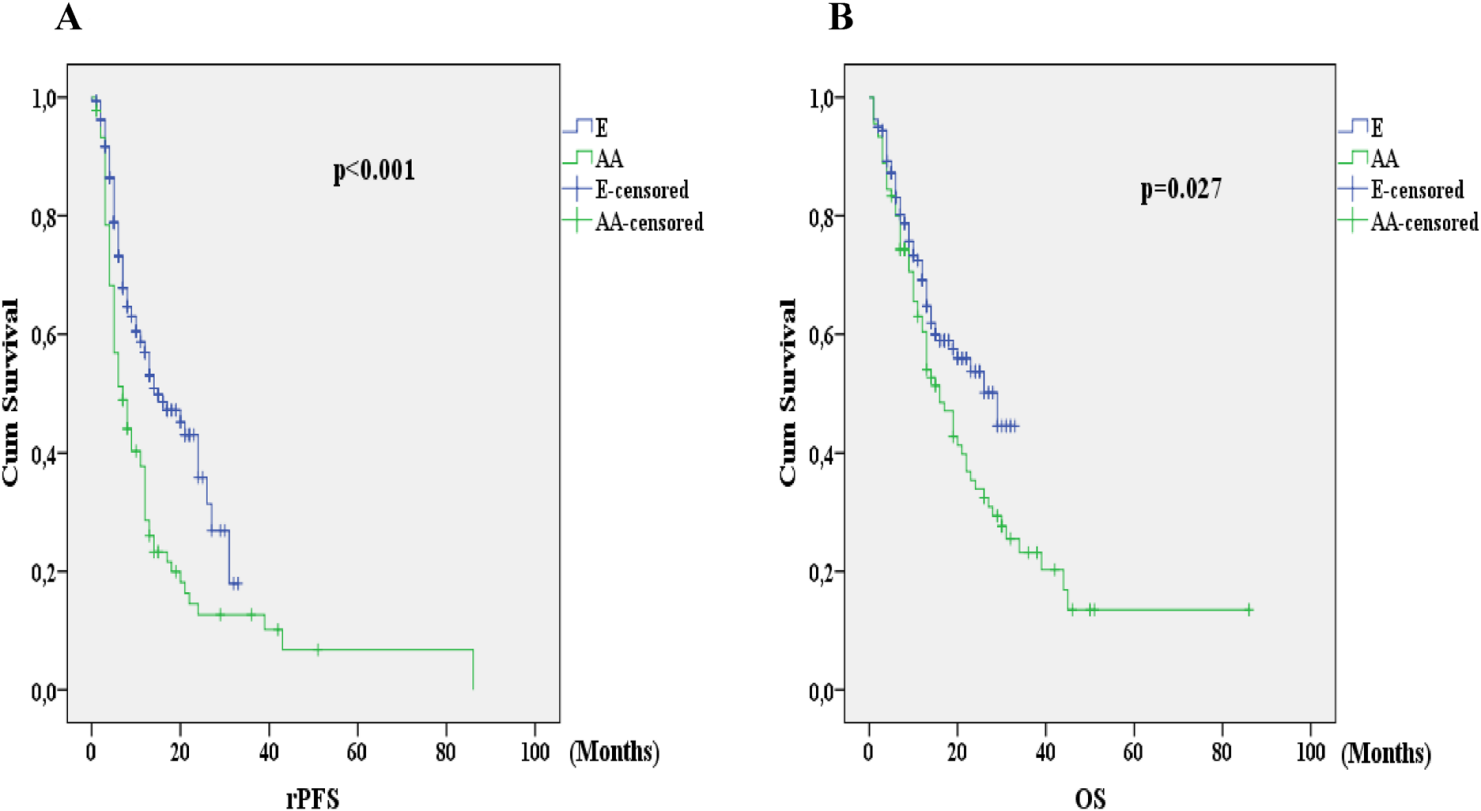
(**A**) Radiological progression free survival in all patients. (**B**) Overall survival in all patients depending on two drugs. *E* enzalutamide, *AA* abiraterone acetate, *rPFS* radiological progression free survival, *OS* overall survival.

A total of 250 patients diagnosed with mCRPC were analyzed. The baseline characteristics of patients are summarized in Table 1. In the AA group, the rate of patients with metastatic disease at the time of diagnosis were significantly higher than that in the E group (p = 0.016). The number of patients with no PSA decline was higher in the AA group than that in the E group, and the proportion of patients with a PSA decline of ≥ 50% was higher in the E group (p = 0.020) (Fig. 1). At the end of the 12th week, progressive disease rate was higher and stable disease rate was lower in the AA group compared to the E group. The median follow-up was 13 months (IQR: 6–21, E: 12 months, AA: 13 months, p = 0.169). During the follow-up period the rate of progression in the AA group (82.2%) was significantly higher than the E group (p < 0.001). Radiological PFS and OS analysis were in favor of E group at a significant level (p < 0.001 and p = 0.027, respectively) (Fig. 2).

**Table 1:** Baseline patient characteristics and comparison between the drug groups.

	All patients (n = 250)	E (n = 160)	AA (n = 90)	p value
Mean age (± SD*), years	72.5 ± 8.5	73.1 ± 8.5	71.7 ± 8.6	0.226
**Age**				
< 75 years, n (%)	139 (55.6)	84 (52.5)	55 (61.1)	0.188
≥ 75 years, n (%)	111 (44.4)	76 (47.5)	35 (38.9)	
Median (IQR*) PSA, ng/mL	67 (22–151)	64 (23–133)	72 (17–210)	0.906
**Gleason, n (%)**				
≤ 7	58 (25.1)	35 (22.7)	23 (29.9)	0.238
≥ 8	173 (74.9)	119 (77.3)	54 (70.1)	
**ECOG-PS, n (%)**				
0–1	156 (62.4)	98 (61.3)	58 (64.4)	0.617
2–3	94 (37.6)	62 (38.8)	32 (35.6)	
**Metastatic sites, n (%)**				
Visceral	52 (20.8)	33 (20.6)	19 (21.1)	0.928
Non-visceral	198 (79.2)	127 (79.4)	71 (78.9)	
**Stage at diagnosis, n (%)**				
Metastatic	153 (61.2)	89 (55.6)	64 (71.1)	0.016
Nonmetastatic	97 (38.8)	71 (44.4)	26 (28.9)	
Pre-docetaxel, n (%)	118 (47.2)	80 (50)	38 (42.2)	0.237
Post-docetaxel, n (%)	132 (52.8)	80 (50)	52 (57.8)	
**PSA decline from baseline, n (%)**				
No decline	51 (22.4)	26 (17.2)	25 (32.5)	0.020
< 50% PSA decline	45 (19.7)	29 (19.2)	16 (20.8)	
≥ 50% PSA decline	132 (57.9)	96 (63.6)	36 (46.8)	
**Radiological response rate, n (%)**				
Complete + Partial remission	92 (37.7)	63 (40.4)	29 (33)	< 0.001
Stable disease	71 (29.1)	56 (35.9)	15 (17)	
Progressive disease	81 (33.2)	37 (23.7)	44 (50)	
**Progression, n (%)**				
Yes	150 (60)	76 (47.5)	74 (82.2)	< 0.001
No	100 (40)	84 (52.5)	16 (17.8)	
Median follow up, months	12.5 (6–20)	12 (6–19)	13 (7–27.3)	0.169
rPFS, months	12 ± 1.2 (9.7–14.3)	15 ± 2.9 (9.2–20.8)	7 ± 1.3 (4.5–9.5)	< 0.001
OS, months	20 ± 2.7 (14.8–25.2)	29 ± 5.8 (17.6–40.4)	16 ± 2.2 (11.7–20.3)	0.027

*E* enzalutamide, *AA* abiraterone acetate, *PSA* total prostate-specific antigen, *ECOG-PS* Eastern Cooperative Oncology Group-Performance Status, *SD* standard deviation *IQR* interquartile range, *rPFS* radiological progression free survival, *OS* overall survival.

*Descriptive results for continuous variables are expressed as mean and standard deviation or as median and interquartile range, depending on the normality of their distribution.

**Figure 3:**
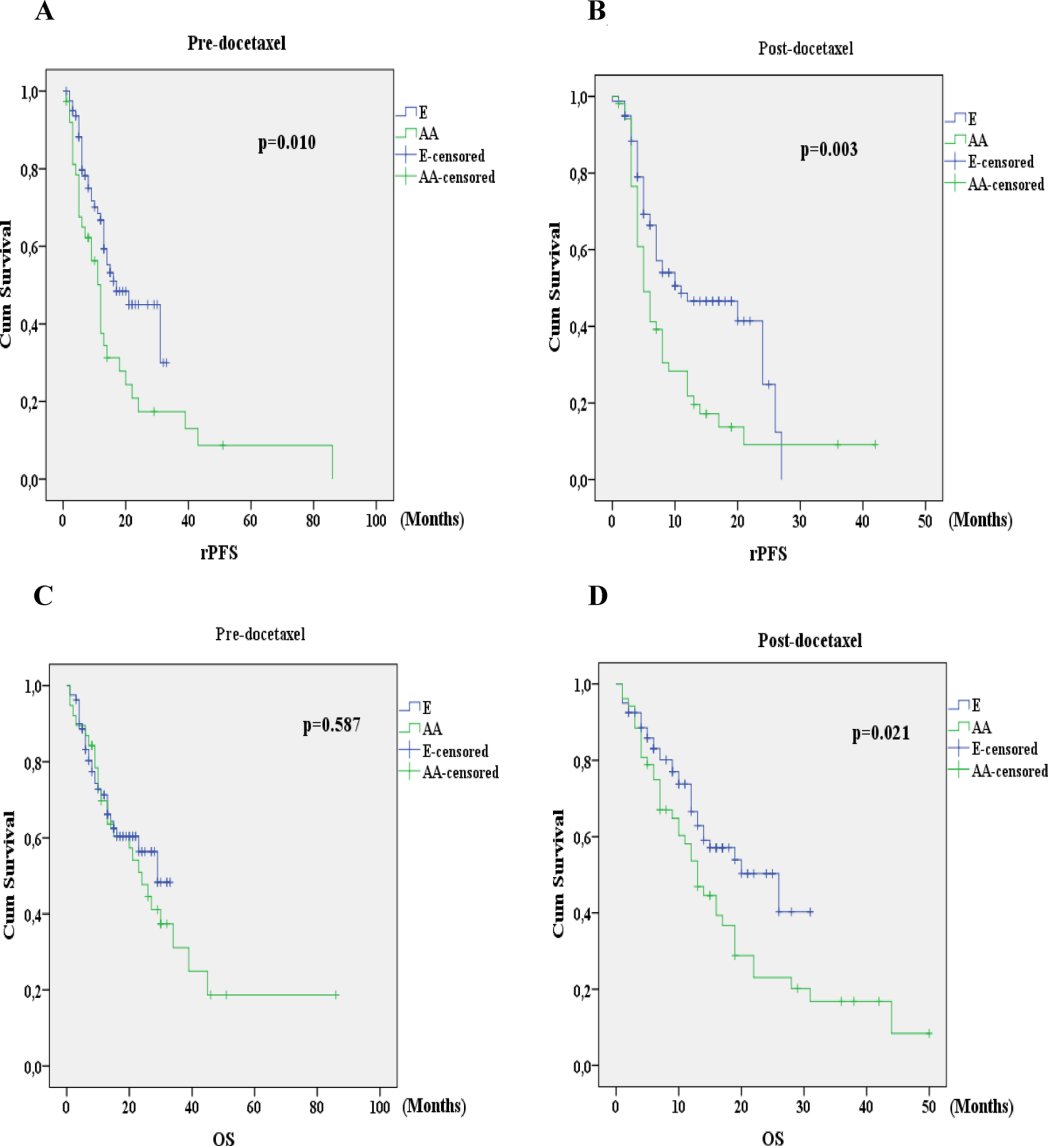
In the pre-docetaxel setting and post-docetaxel setting (**A,B**) Comparison of radiological progression free survival (**C,D**) Comparison of overall survival between two drugs. *E* enzalutamide, *AA* abiraterone acetate, *rPFS* radiological progression free survival, *OS* overall survival.

The subgroup analysis results of the pre-docetaxel and post-docetaxel settings are demonstrated in Table 2. In the E group, rPFS was significantly longer than that in the AA group in both pre-docetaxel and post-docetaxel settings. OS was similar in the pre-docetaxel setting; but in the post-docetaxel setting, E group had a significantly longer OS than the AA group (Fig. 3). In E and AA groups 23.8% (n = 19) and 76.3% (n = 61) and 11.5% (n = 6) and 88% (n = 46) of the patients received combination therapy and ADT alone (p = 0.062) in metastatic hormone sensitive setting, respectively. Previous treatments other than docetaxel included orchiectomy in 6.4% of the patients and luteinizing hormone releasing hormone agonists in 93.6%. Radical prostatectomy was performed in 5.6% of the patients, while 5.6% had undergone definitive radiotherapy.

**Table 2:** The subgroup analysis results of the pre-docetaxel and post-docetaxel setting.

	Pre-docetaxel			Post-docetaxel		
	E (n = 80)	AA (n = 38)	P value	E (n = 80)	AA (n = 52)	p value
Median follow-up (IQR*), months	13 (6.0–20.7)	17.5 (8.7–30.0)	0.028	12 (6–17)	12.5 (6.2–19)	0.498
Progression**, n (%)	34 (42.5)	31 (81.6)	< 0.001	42 (52.5)	43 (82.7)	< 0.001
rPFS, months	17 ± 4.6 (8–26)	12 ± 1.3 (9.4–14.6)	0.010	11 ± 5.1 (1.1–20.9)	5 ± 0.7 (3.6–6.4)	0.003
OS, months	29 ± 3.0 (23.0–35.0)	24 ± 4.0 (16.0–32.0)	0.587	26 ± 7.0 (12.3–39.7)	13 ± 1.6 (9.8–16.2)	0.021

*IQR* interquartile range, *E* enzalutamide, *AA* abiraterone acetate, *rPFS* radiological progression free survival, *OS* overall survival.

*Descriptive results for continuous variables are expressed as mean and standard deviation or as median and interquartile range, depending on the normality of their distribution.

**Progression rate during the follow-up period.

The univariate analysis results for the factors affecting rPFS and OS are summarized in Table 3. In the univariate analysis, the treatment agent (E and AA) significantly predicted both rPFS and OS. The other factors that significantly predicted rPFS were age, pre- or post-docetaxel setting and PSA decline rate. Besides, the factors that significantly affected OS were pre- or post-docetaxel setting and PSA decline rate. Multivariate Cox regression analysis was performed for parameters that had a significant or near-significant effect (p < 0.250) on rPFS and OS (Table 4). Multivariate analysis results showed that age, treatment agent, PSA decline rate and metastatic sites were independently associated with rPFS. No factor was detected as an independent predictor of OS.

**Table 3:** Univariate analysis of rPFS and OS in all patient population.

	Univariate rPFS Median ± SE (95% CI)	P value	Univariate OS Median ± SE (95% CI)	p value
**Drug**				
E	15 ± 2.9 (9.2–20.8)	< 0.001	29 ± 5.8 (17.6–40.4)	0.027
AA	7 ± 1.3 (4.5–9.5)		16 ± 2.2 (11.7–20.3)	
**Age, years**				
< 75	8 ± 1.6 (4.9–11.1)	0.010	19 ± 2.3 (14.5–23.5)	0.516
≥ 75	13 ± 1.3 (10.5–15.5)		26 ± 3.7 (18.8–33.2)	
**Gleason**				
≤ 7	14 ± 3.7 (6.8–21.2)	0.320	26 ± 8.1 (10.1–41.9)	0.632
≥ 8	11 ± 1.5 (8.1–13.9)		20 ± 2.5 (15–25)	
**ECOG-PS**				
0–1	11 ± 1.3 (8.5–13.5)	0.894	22 ± 3.3 (15.6–28.4)	0.063
2–3			14 ± 2.8 (8.4–19.6)	
**Metastatic sites**				
Visceral	7 ± 2.3 (2.5–11.5)	0.090	22 ± 7.9 (6.5–37.5)	0.932
Non-visceral	12 ± 0.9 (10.2–13.8)		20 ± 2.8 (14.5–25.5)	
**Stage at diagnosis**				
Metastatic	11 ± 1.4 (8.2–13.8)	0.670	19 ± 2.6 (13.9–24.1)	0.461
Nonmetastatic	13 ± 1.8 (9.5–16.5)		26 ± 3.4 (19.3–32.7)	
Pre-docetaxel	14 ± 1.7 (10.8–17.2)	0.002	26 ± 3.3 (19.4–32.6)	0.048
Post-docetaxel	7 ± 0.8 (5.5–8.5)		16 ± 2.1 (11.9–20.1)	
**PSA decline from baseline**				
No decline	5 ± 0.4 (4.1–5.9)	< 0.001*	11 ± 1.7 (7.6–14.4)	< 0.001**
< 50% PSA decline	7 ± 3.2 (0.6–13.4)		15 ± 2 (11.1–18.9)	
≥ 50% PSA decline	18 ± 2.3 (13.5–22.5)		29 ± 3.9 (21.4–36.6)	
**Radiological response rate, n (%)**				
CR + PR	20 ± 3.1 (13.8–26.2)	0.871	34 ± 4 (26.2–41.8)	0.165
Stable disease	26 ± 8.1 (10.1–41.9)		26 ± 3.8 (18.5–33.5)	

*SE* standard error, *CI* confidence interval, *E* enzalutamide, *AA* abiraterone acetate, *PSA* prostate-specific antigen, *ECOG-PS* Eastern Cooperative Oncology Group-Performance Status, *rPFS* radiological progression free survival, *OS* overall survival, *CR* complete remission, *PR* partial remission.

*There was a significant difference between No decline vs. < 50% PSA decline, No decline vs. > 50% PSA decline, and < 50% PSA decline vs. > 50% PSA decline.

**There was a significant difference between No decline vs. > 50% PSA decline, < 50% PSA decline vs. > 50% PSA decline, but no significant difference between No decline vs. < 50% PSA decline.

**Table 4:** Multivariate Cox regression analysis of rPFS and OS in all patient population.

	Multivariate analysis of rPFS		
	HR	95% CI lower–upper	p value
Treatment (E vs. AA)	1.54	1.10–2.20	0.015
Age (≥ 75 years vs. < 75 years)	1.55	1.05–2.27	0.026
**PSA decline from baseline (reference; ≥ 50% PSA decline)**			
< 50% PSA decline	1.72	1.10–2.74	0.020
No decline	3.40	2.27–5.06	< 0.001
Metastatic sites (nonvisceral vs. visceral)	1.34	0.91–2.00	0.135
Pre-docetaxel vs. post-docetaxel	1.12	0.60–2.11	0.714
Treatment (E vs. AA)	1.02	0.54–1.90	0.960
ECOG PS (0–1 vs. 2–3)	1.60	0.90–2.90	0.128
**PSA decline from baseline (reference; ≥ 50% PSA decline)**			
< 50% PSA decline	1.40	0.54–3.60	0.495
No decline	0.90	0.40–1.91	0.733
Radiological response (complete + partial vs. stable)	1.60	0.85–3.02	0.145
Pre-docetaxel vs. post-docetaxel	1.12	0.50–0.164	0.709

*HR* hazard ratio, *CI* confidence interval, *E* enzalutamide, *AA* abiraterone acetate, *PSA* prostate-specific antigen, *ECOG-PS* Eastern Cooperative Oncology Group-Performance Status, *rPFS* radiological progression free survival, *OS* overall survival.

## Discussion

In this study, we mainly aimed to examine whether there is a difference between rPFS and OS of mCRPC patients treated with E and AA. In addition, we evaluated prognostic factors affecting rPFS and OS in this patient group. Although E was statistically significantly superior to AA in terms of rPFS and OS, it didn’t provide a significant reduction in death risk compared to AA. In all patients, being < 75 years of age, PSA decline of < 50% at 12 weeks of treatment were found to be poor risk factors for rPFS. In our study, rPFS and OS were 12 months and 20 months in the entire cohort, 15 months and 29 months in the E group, and 7 months and 16 months in the AA group, respectively (E vs. AA; p < 0.001 for rPFS, p = 0.027 for OS).

In COU AA-301(post-docetaxel) and COU AA-302 (pre-docetaxel) studies, the median rPFS was 8.5 months and 16.3 months, respectively. The respective median OS was 15.8 months and 34.7 months in the AA groups, with superiority over placebo for both^4,8^. In our study, AA treatment in post-docetaxel and pre-docetaxel settings resulted in a rPFS of 5 months and 12 months, and an OS of 13 months and 24 months, respectively. The shorter rPFS and OS compared to the COU AA301 and 302 studies may be due to the shorter follow-up time in our study. Also, clinical outcomes in our study were inferior than those reported in COU AA-301 and 302 studies. The inferiority in terms of survival in the current study is likely to be due to inclusion of patients with more advanced disease, including those with visceral metastases. Furthermore, performance status, hemoglobin level, and presence of pain, which are known to be the most important determinants of survival in CRPC patients, were not taken into consideration while enrolling patients. CAO AA-301 and 302 were randomized phase 3 trials, in which the proportion of patients with visceral metastases were lower than those observed in real life settings; in fact, patients with visceral metastases were even not included in COU AA-302. Also, the inclusion criteria for these studies were more strict.

In the phase III, randomized AFFIRM trial, the median OS and the time to PSA progression were 18.4 months and 8.3 months in patients who received E in the post-docetaxel setting, respectively^9^. In the PREVAIL study, which was terminated early due to the clear superiority of pre-docetaxel E treatment in terms of rPFS (20 months vs. 5.4 months) over placebo. Median OS was 35.3 and 31.3 months in the treatment and placebo arms, respectively^10^. In our study, E given in post-docetaxel and pre-docetaxel settings revealed a rPFS of 11 months and 17 months, an OS of 26 months and 29 months, respectively. Although the median follow-up time was shorter when compared to the studies mentioned above, the results were consistent with the literature. Even, our post-docetaxel rPFS and OS were longer than the AFFIRM study.

Simon et al. compared first line AA, E and docetaxel activities in a multi-center, retrospective study of 1874 patients with mCRPC. The median time to progression in the AA, E and docetaxel groups was 9.6, 10.3, and 7.6 months, respectively; the median OS was 27.1, 27.1, and 27.9 months, respectively^11^. In our study, rPFS was 12 and 17 months in the AA and E groups, and the median OS was 24 and 29 months, respectively. Oyman et al. retrospectively evaluated CT-naive and post-CT mCRPC patients who received AA. Median rPFS was 10.1 months in all patients, 10.1 months in the CT-naive group, and 9.7 months in the post-CT group (p = 0.808). The median OS was 17.3 months in all patients, 12.7 months in the CT-naive group, and 29.4 months in the post-CT groups (p = 0.236). While the numerical superiority in the results of our study was in patients who received pre-CT AA, the results of Oyman et al. were in favor of post-CT AA^12^. Marret et al. evaluated the efficacy of AA in 93 patients with mCRPC; the median duration of treatment with AA was 12.7 months and 7.5 months; median OS was 36.4 months and 13.4 months in pre-docetaxel (n = 33) and post-docetaxel (n = 58) settings, respectively. Similar results were obtained in our study^13^. Another real-world study evaluated 110 patients with mCRPC who were treated with AA. Of the patients, 58 and 52 received AA in prechemotherapy (preCT) and postchemotherapy (postCT) settings, respectively. Median PFS was 15.5 and 6.4 months, and OS was 18.1 and 6.7 months for preCT and postCT groups, respectively. Similar to our study, the factor affecting PFS and OS was a decline of > 50% in PSA levels in the first 3 months. Survival was significantly lower in patients with visceral metastasis^14^.

Nadal et al. examined 107 patients who were treated with E. Of the patients, 60 were pretreated with docetaxel and 47 were docetaxel-naive. Median PFS was superior in the docetaxel naive group (p < 0.0001). They claimed that E activity was lower in patients who had previously received docetaxel CT and thought that there might be cross resistance between docetaxel and E. The follow-up period in the study of Nadal et al. was shorter than that in our study^15^. In a Japanese retrospective study about the treatment efficacy, safety profile, and prognostic factors of E, 184 patients with non-mCRPC and mCRPC were analyzed; 44 (23.9%) non-mCRPC patients, 89 (48.4%) docetaxel-naive mCRPC patients, and 51 mCRPC patients pretreated with docetaxel (27.7%) mCRPC patients underwent E therapy. The median PSA PFS was 16.5 and 7.0 months, and overall survival was 59.8 and 30.4 months for docetaxel-naive and for docetaxel-pretreated mCRPC patients, respectively. Multivariate analysis identified that the predictive factor for a shorter OS was 4-week PSA decline < 50%. This study had a relatively longer observation period with a median follow-up of 41.3 months, than the other retrospective studies and our study^16^.

The other retrospective studies comparing E and AA in a design similar to our study were reviewed. Al-Ali et al. analyzed 457 patients with CRPC who received AA and/or E in preCT and postCT settings. The median OS of the entire cohort was 21 months, 15 months for the AA group, 24 months for the E group, 26 months for the sequence group, and 10 months for the sequence group after switching. Median OS in the pre-CT setting was 25 months (mean: 21.5 ± 1.1 months) in the entire cohort, 18 months in AA group (mean: 18.9 ± 1.5 months) and 17 months in E treatment group (mean: 18.2 ± 1.9 months). In the post-CT setting, the median OS was 14 months in the AA group (mean: 15.8 ± 0.9 months), 19 months in the E group (mean: 17.2 ± 1.4 months) and 25 months in the sequence group (mean: 22.7 ± 0.8 months)^17^. In the study of Al-Ali et al., OS was shorter than our study and the other pilot studies in mCRPC patients treated with AA and E. Miyake et al. compared the efficacy of AA and E in mCRPC patients in pre-CT setting. The study included 280 mCRPC patients, of the patients 113 and 167 were receiving AA and E, respectively. In the E group, PSA response rate and PSA PFS were significantly higher than that in the AA group. Duration of ADT treatment and ECOG PS for the AA group, age and ECOG-PS for the E group, and ECOG-PS for the overall patients were identified as the independent predictors of PSA PFS. The rate of patients with grade ≥ 3 side effects in the E group (11.4%) was significantly higher than that in the AA group (4.4%)^18^. In a meta-analysis, Wang et al. compared the clinical efficacy and safety of AA and E in mCRPC patients on the results of 14 cohort studies including 3469 patients. Pooled results demonstrated that E was more effective than AA for patients with mCRPC, however was related with a significantly elevated risk of side effects, particularly fatigue. Comparisons for PFS were mentioned in 3 studies (n = 386) and comparisons for OS in 4 studies (n = 774)^19^. Similar to our results Miyake et al. found a significant difference in PFS between E and AA treatment in docetaxel-naive mCRPC patients (median PFS, E vs. AA; 11.6 vs. 9.0 months, p = 0.014). Additionally, in 4 studies, the two drugs were not different in terms of OS. Norris et al. compared mCRPC patients treated with AA or E. Similar to our study, more patients in the E (51%) than the AA (36%) group had a > 50% PSA decline (p = 0.031). However, there was no significant difference between the two groups in terms of OS (OS was 15.3 months vs. 22.2 months, AA vs. E, p = 0.913) and in the time-to-treatment failure (p = 0.464)^20^.

Due to the shorter follow-up time compared to other studies, rPFS and OS were relatively shorter in our study when compared to the results of other E and AA studies. Our study was retrospective, but as the patient groups had similar clinicopathological features, the results can be used to compare the efficacy of the two drugs in mCRPC patients. There is currently no prospective study in the literature similar to the design of this study. We suggest that the reasons why E was found to be significantly superior to AA in terms of rPFS and OS, were that the rate of metastasis at the time of diagnosis was significantly higher in the AA group (E vs. AA, 55.6 vs. 71.1 p = 0.016) and the PSA decline rates were lower in the AA group (p = 0.020). When the PSA decrease rates were examined, it was found that only ≥ 50% PSA decrease in the AA group had a significant effect on both rPFS and OS. Also, a prognostic difference is known to exist between combination therapy (ADT with docetaxel) and ADT monotherapy in metastatic hormone sensitive settings. There was a numerical superiority for the combination therapy in group E (23.8% in the E group vs 11.5% in the AA group) in metastatic hormone sensitive setting. Therefore, a survival effect might have occurred in the post-docetaxel group. In the real-world studies performed with E, the most noticeable side effect was fatigue, which was higher than that found in phase 3 prospective trials. Since our study lacked side effect data, we could not give any results on this topic.

With the recent use of E and AA in hormone sensitive patients, the question is whether our real-world data will be compatible with the results of randomized prospective studies in the literature. Therefore, real-life parameters affecting rPFS and OS in our study and other retrospective studies will guide us to discover new indications for these drugs. Since the rPFS and OS curves for the patients who progressed or died have not yet reached a median in the E group, the data is still immature. Real-life studies with longer follow-up are needed.
